# Reviewing clinical considerations and guideline recommendations of C1 inhibitor prophylaxis for hereditary angioedema

**DOI:** 10.1002/clt2.12092

**Published:** 2022-01-18

**Authors:** John Anderson, Njeri Maina

**Affiliations:** ^1^ Clinical Research Center of Alabama, AllerVie Health Birmingham Alabama USA; ^2^ Alabama Allergy and Asthma Center, AllerVie Health Birmingham Alabama USA

**Keywords:** bradykinin, C1 inhibitor, hereditary angioedema, kallikrein, long‐term prophylaxis

## Abstract

**Background:**

Hereditary angioedema (HAE), a rare disease that is characterized by painful and recurring non‐allergic swelling episodes, is caused by the deficiency or dysfunction of C1 inhibitor (C1INH) protein. A comprehensive HAE management plan may require long‐term prophylaxis (LTP) in addition to on‐demand treatment to help “normalize” patients' lives so that they may fully engage in work, school, family, and leisure activities.

**Aim:**

The main objective of this narrative review is to provide an overview of updated guideline recommendations specific to LTP of HAE and discuss clinical considerations and pharmacologic management options, with a focus on C1INH.

**Materials and Methods:**

The authors reviewed relevant HAE literature for current recommendations regarding LTP and the role of C1NH.

**Results:**

Acute HAE attacks are treated with on‐demand medication; however, there is a consensus that LTP should routinely be considered for risk reduction and prevention of future episodes. The 2017 World Allergy Organization/European Academy of Allergy and Clinical Immunology guidelines recommend that all patients with HAE be evaluated for LTP routinely and the 2020 HAE Association (HAEA) guidelines emphasize that the decision to use LTP should not be based on rigid criteria, but rather should be based on individual patient needs. Both guidelines recommend C1INH as first‐line/preferred therapy for LTP in a range of patient types including adults, children/adolescents, and pregnant/lactating patients. The HAEA also recommends the kallikrein inhibitor, lanadelumab, as a first‐line option for LTP. HAE pathway‐specific agents for LTP have not been associated with notable safety concerns.

**Discussion:**

Plasma‐derived C1INH has been available for 40+ years in Europe and impacts multiple targets within the HAE pathway. C1INH has been used for on‐demand treatment and LTP. A subcutaneous formulation of plasma‐derived C1INH is approved for LTP and produces functional C1INH activity levels consistently above the threshold needed for protection from HAE attacks. Other pathway‐specific options for LTP include the plasma kallikrein inhibitors, lanadelumab‐flyo and berotralstat, approved for adults and pediatric patients aged ≥12 years. C1INH is approved for adults and pediatric patients aged ≥6 years.

**Conclusion:**

Assessing the need for LTP is vital in the ongoing dialogue between clinicians and patients, as both disease‐related factors and patient preferences may change over time. Among available options for LTP, plasma‐derived C1INH is the broadly recommended first‐line option for LTP in patients with HAE, including pregnant/lactating women and pediatric patients (≥6 years).

## 1 INTRODUCTION

Hereditary angioedema (HAE) is caused by either a deficiency of C1 inhibitor (C1INH) protein (HAE Type I) or dysfunction of C1INH (HAE Type II).^1^, ^2^ Endogenous C1INH has a major role in regulating the complement and contact pathways, an important role in coagulation, and, under certain circumstances, impacts the fibrinolytic pathway.^2^, ^3^ In patients with HAE, deficiency in the levels and/or function of endogenous C1INH leads to production of excess bradykinin that underlies the recurrent episodes of swelling.^1^, ^4^ Most patients experience painful episodes of angioedema including recurrent skin swelling, recurrent abdominal pain episodes, and rarely occurring laryngeal edema.^5^ Angioedema associated with HAE is considered to be non‐allergic, since the underlying mechanism is bradykinin‐mediated and distinct from the more familiar allergic, histamine‐mediated angioedema.

**FIGURE 1:**
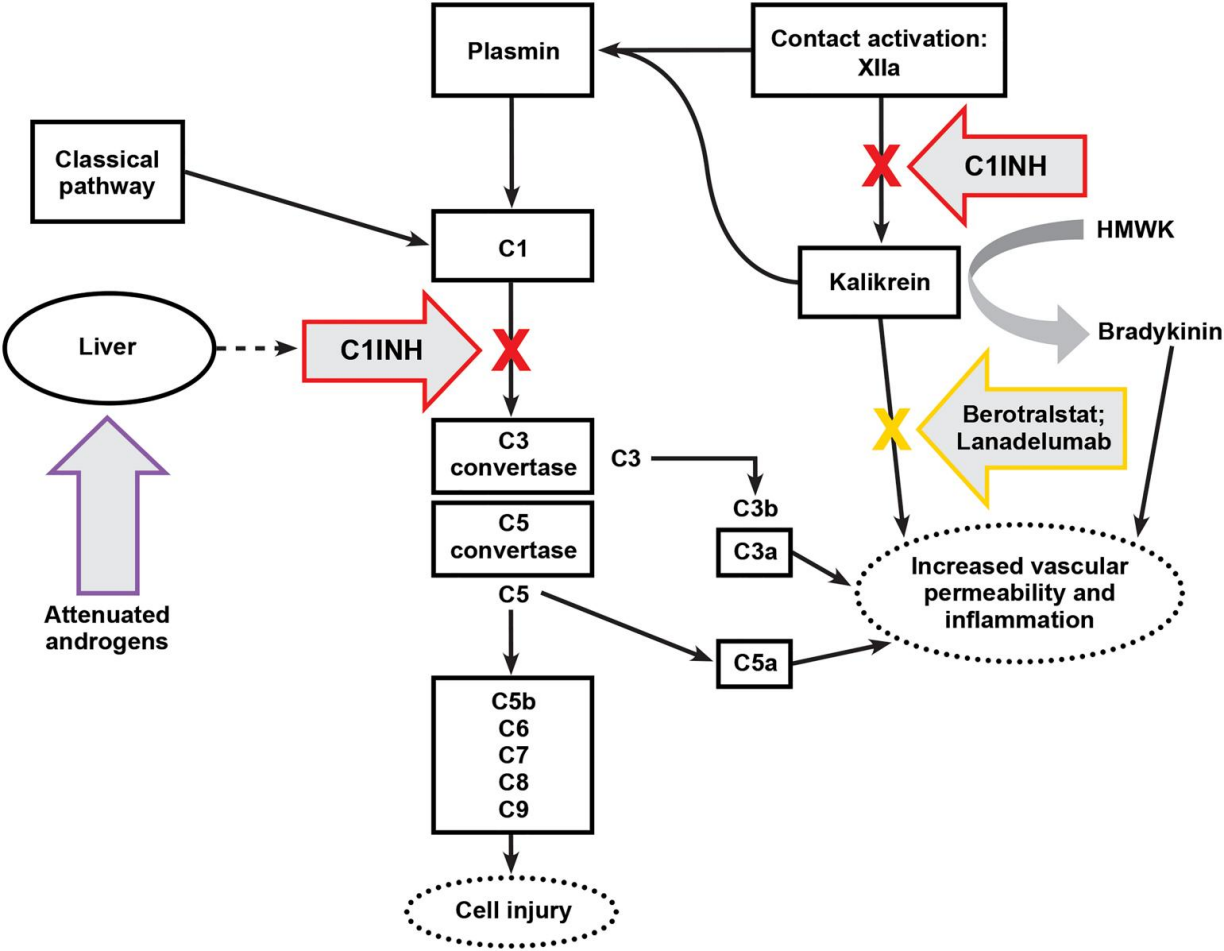
Mechanisms of action for therapies used in LTP of HAE.^6^, ^7^ Mechanisms of action for therapeutic agents in treating or preventing HAE. Multiple pathways are capable of complement activation and generating inflammatory mediators including complement anaphylatoxins C3a and, more important, C5a. Activation of the final complement cascade produces a membrane attack complex that produces cellular injury. Angioedema occurs after tissue injury from multiple causes. Tissue injury can activate contact activation (Hageman factor or factor XII) to generate kallikrein from prekallikrein, its precursor. Kallikrein in turn generates and activates plasmin from plasminogen, and plasmin can directly activate the C1 esterase complex to initiate complement activation. Under normal circumstances, C1INH functions to inhibit both complement activation and, to a lesser extent, modulate contact activation. In HAE‐C1INH, because of quantitative or qualitative defective C1INH, the pathway proceeds unchecked, generating mediators that increase capillary permeability to produce angioedema. Therapeutic approaches for LTP of HAE include restoring C1INH levels with C1INH replacement or inhibiting kallikrein with lanadelumab or berotralstat. Attenuated androgens have been used as second‐line options to increase liver synthesis of C1INH. Figure and description has been adapted from Wahn et al.^6^ under the Creative Commons CC‐BY license, which permits unrestricted use, distribution, and reproduction in any medium. C1INH, C1 inhibitor; HAE, hereditary angioedema; HMWK, high‐molecular‐weight kininogen; LTP, long‐term prophylaxis

**FIGURE 2:**
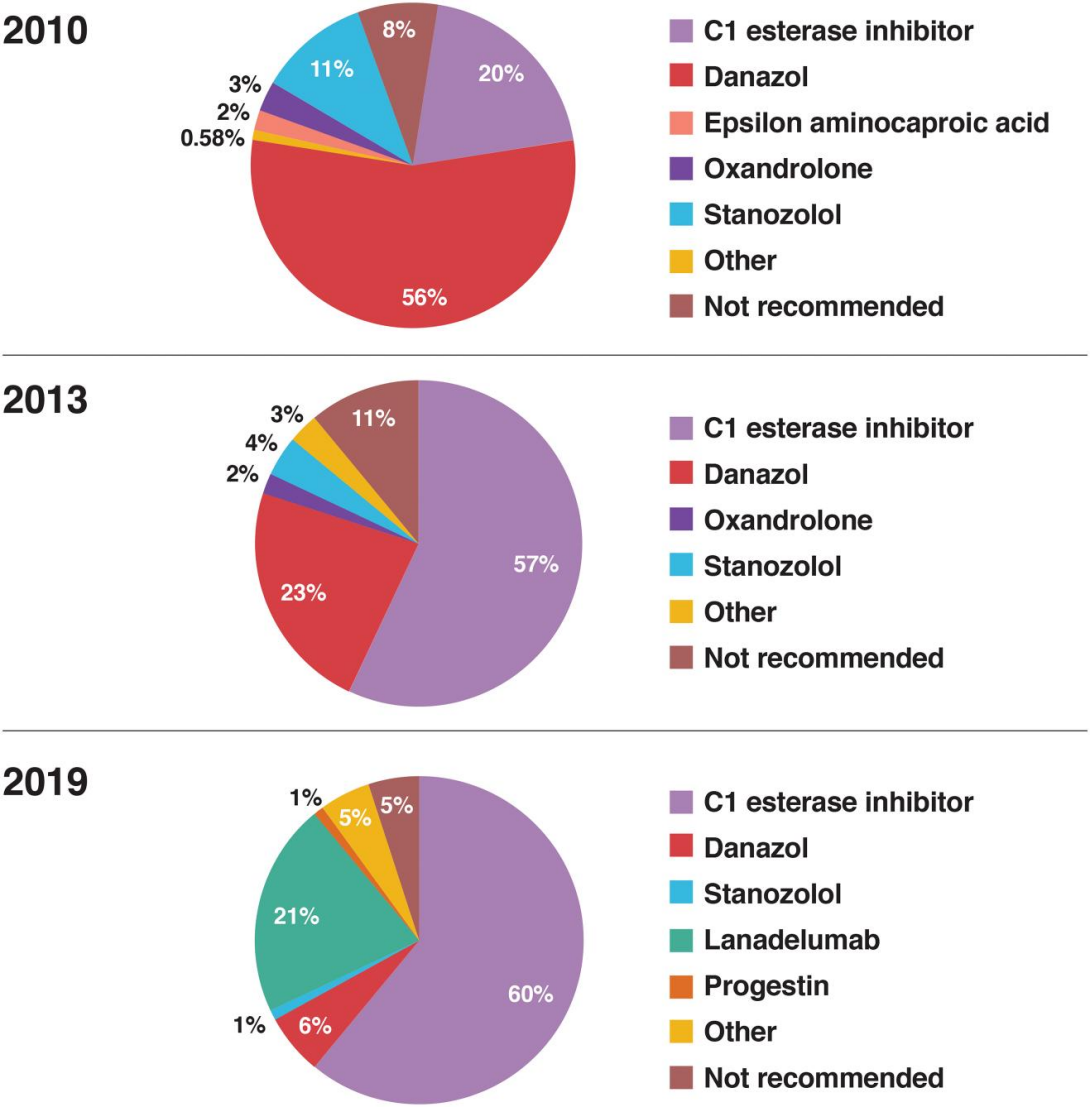
Long‐term HAE prophylaxis and medications reported as used “most frequently”.^8^ Proportion of physicians reporting HAE LTP and medications used “most frequently.” Over the past decade, reported use of pathway‐specific options has increased, while use of anabolic steroids has decreased. The figure is reproduced with permission from Riedl et al.^8^ under the Creative Commons CC‐BY license, which permits unrestricted use, distribution, and reproduction in any medium. HAE, hereditary angioedema; LTP, long‐term prophylaxis

Figure 1 depicts the pathophysiology of the HAE pathway. Many HAE pathway‐based treatments are now available and have become the treatments of choice over the past decade, while previously popular therapies, such as androgens, have fallen out of favor (Figure 2).^8^ Direct replacement of naturally occurring C1INH with plasma‐derived C1INH (hereafter referred to as C1INH) or recombinant C1INH impacts multiple targets within the HAE pathway (Figure 1).^6^, ^9^, ^10^ Other HAE pathway‐based treatments target either plasma kallikrein or bradykinin specifically.^7^ For treatment of acute attacks, it is recommended that all patients with HAE not only have on‐demand treatment for two attacks, but also carry their on‐demand treatment, whatever it may be, at all times.^11^ In addition to having appropriate on‐demand medication for acute attacks, there is a consensus that long‐term prophylaxis (LTP) should be considered for risk reduction and prevention of future episodes.^12^ Thus, for many patients, comprehensive management of HAE may be comprised of on‐demand rescue medications as well as LTP. Of note, for patients who have an increased risk of HAE attacks associated with known triggers (e.g., invasive dental, medical, or surgical procedures and/or stressful life events), short‐term prophylaxis (STP) with a single dose of C1INH (1–12 h prior to the stressor) or a short course of anabolic steroids (started 5–7 days before the event and continued for 2–5 days after) may be appropriate^13^; however, the current review is focused on LTP.

This narrative review provides an overview of updated guideline recommendations specific to LTP of HAE and discusses pharmacologic management options, with a focus on C1INH as it is recommended as first‐line treatment in children, adults, and specialized populations. In addition, clinical practice considerations regarding LTP will be shared.

## 2 TARGETING THE HAE PATHWAY FOR LTP OF HAE

### 2.1 Bradykinin inhibition

No pathway‐based products targeting bradykinin are approved for LTP. The bradykinin B2 receptor antagonist, icatibant, is indicated for treatment of acute HAE only.^14^

### 2.2 C1INH replacement

C1INH has been available in Europe for over 40 years,^15^ and intravenous (IV) formulations of C1INH, BERINERT^®^ (CSL Behring, LLC) and CINRYZE^®^ (Shire ViroPharma Biologics, Inc.) were approved in the US over a decade ago for acute treatment of HAE attacks (BERINERT) and routine prophylaxis (CINRYZE) in patients with HAE.^16^, ^17^, ^18^ A recombinant IV C1INH formulation (RUCONEST^®^; Pharming Healthcare, Inc.) is also approved for on‐demand treatment.^19^

The efficacy of C1INH(IV) (Shire ViroPharma Biologics, Inc.) for LTP was demonstrated in a 24‐week cross‐over study (NCT01005888) of patients with HAE who experienced ≥2 attacks/month.^20^ Attack frequency was reduced by approximately 50% with C1INH(IV) 1000 U given every 3–4 days compared with placebo (12.7 vs. 6.3 attacks per month; mean difference in attack rates 6.47 attacks/month; 95% CI, 4.21–8.73; *p* < 0.001). In a subsequent open‐label study that included patients from the original 24‐week cross‐over study, the efficacy of C1INH(IV) was demonstrated over a longer time frame (median duration of ∼35 weeks, ranging from >24–72 weeks).^21^ Participants in the long‐term study experienced a >90% reduction in HAE attacks, from a median of 3 attacks per month at screening to 0.19 attacks per month while on C1INH(IV) for LTP, and no notable safety issues were associated with the treatment. C1INH(IV) doses up to 2500 U (not exceeding 100 U/kg) every 3 or 4 days may be considered based on individual patient response^17^ (e.g., for patients experiencing breakthrough attacks with the 1000 U dose). LTP labeling for C1INH(IV) has been expanded from adults to patients aged ≥6 years.^17^, ^18^

A subcutaneous (SC) formulation of C1INH, HAEGARDA^®^ (CSL Behring, Marburg, Germany), labeled as Berinert 3000 in the EU, was subsequently approved for the routine prevention of HAE attacks, initially in adolescents and adults^22^ (EU labeling continues to be for “adolescents” and adults only^23^) and more recently expanded to patients aged ≥6 years in the United States.^24^

The pivotal 16‐week COMPACT phase three study (NCT01912456) assessed the C1INH(SC) formulation for LTP in patients aged ≥12 years who had HAE type 1 or 2 and experienced ≥2 attacks/month.^25^ Patients randomized to the C1INH(SC) 60 IU/kg twice‐weekly group had a significantly lower rate of HAE attacks/month compared with those randomized to the placebo group (mean [95% confidence interval]: 0.52 [0.00–1.04] vs. 4.03 [3.51–4.55]; *p* < 0.001). The majority (90%) of patients in the C1INH(SC) 60 IU/kg group had *a* ≥ 50% reduction in HAE attacks. A prespecified post‐hoc analysis of COMPACT found onset of attack prevention within 2 weeks of C1INH(SC) initiation.^26^ Another post‐hoc analysis of the COMPACT that focused on prespecified exploratory health‐related quality of life (HRQoL) parameters found that C1INH(SC) improved anxiety, work productivity, and activity impairment compared with on‐demand treatment.^27^

In the long‐term open‐label extension of COMPACT (NCT02316353), C1INH(SC) demonstrated sustained reductions in HAE attacks, symptoms, and the need for rescue medication without emergence of safety concerns over a median duration of 52.6 weeks. The C1INH(SC) 60 IU/kg twice weekly group had a median time‐normalized attack rate of 1 HAE attack per year.^28^ Considering the COMPACT HRQoL analysis findings of improved anxiety and increased work productivity, together with the open‐label extension findings of a safe and sustained effect, LTP with C1INH(SC) may allow patients to become free of HAE disease symptoms and enable them to regain an active and productive lifestyle.^27^, ^28^

**FIGURE 3:**
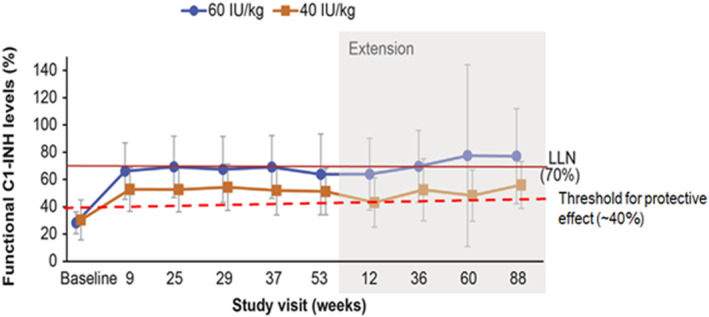
Improvement in functional C1INH levels with C1INH(SC) (phase 3 COMPACT and OLE).^28^ Pharmacodynamic findings over the course of the COMPACT study and OLE. Mean (dots) and SD (vertical lines) of C1INH functional activity; gray boxed area indicates the extended study period. This figure is reproduced, with slight alterations (as described in the following sentences), from *J Allergy Clin Immunl Pract*, *7*(6), Craig T et al., Long‐term outcomes with subcutaneous C1‐inhibitor replacement therapy for prevention of hereditary angioedema attacks, 1793‐1802.e2, 2019, with permission from Elsevier. LLN taken from Tarzi et al.^31^ Threshold for protective effect based on Spath et al.^29^ C1INH, C1 inhibitor; C1INH(SC), subcutaneous C1INH; IU, international units; LLN, lower limit of normal; OLE, open‐label extension

The SC formulation filled a previously unmet need related to C1INH replacement: the pharmacodynamic profile of C1INH(SC) is characterized by steady state levels of functional C1INH activity above the ∼40% threshold required for adequate protection from HAE attacks—this is distinct from the IV formulation, which is characterized by more variable peaks and troughs, with the trough levels below the ∼40% threshold.^29^, ^30^ Pharmacodynamic data from the COMPACT study show that the mean C1INH functional activity increased from ∼30% at baseline (prior to initiation of LTP) to >65% (approximating the lower limit of the normal range of ∼70%) in the 60 IU/kg twice‐weekly group; this increase of C1INH functional activity to well above the ∼40% threshold for protective effect was sustained in the extension period of up to 88 additional weeks (Figure 3).^28^ These results were consistent with data from an earlier phase 2 study of C1INH, which showed dose‐dependent increases in trough plasma levels of functional C1INH activity to be above the threshold for a protective therapeutic effect.^30^ Of interest, a post‐hoc analysis of COMPACT found that, among patients who were using the IV formulation of C1INH as routine prophylaxis pre‐study, switching to the SC formulation of C1INH produced a clinically meaningful ∼50% mean reduction in HAE attacks.^32^ Another important point is that available data support that rescue medications remain effective in patients on LTP for HAE. For example, in the COMPACT phase 3 study, use of C1INH(SC) for LTP did not diminish the effect of on‐demand treatments, such as C1INH(IV), icatibant, or ecallantide (i.e., most patients needed just 1 dose of rescue medication).^25^, ^28^

### 2.3 Plasma kallikrein inhibition

C1INH inhibits multiple targets in the contact system, including plasma kallikrein. The first drug specifically targeting plasma kallikrein, the SC kallikrein inhibitor ecallantide (KALBITOR^®^; Takeda Dyax Corp), was developed for on‐demand treatment and approved in the US, though not yet in the EU.^33^ More recently, 2 plasma kallikrein inhibitors have been developed for LTP.

Lanadelumab‐flyo (TAKHZYRO^TM^; Shire) is a recombinant, human monoclonal antibody long‐acting inhibitor of kallikrein^34^ approved as prophylaxis to prevent HAE attacks in patients aged ≥12 years.^35^, ^36^ Efficacy of lanadelumab for HAE type 1 and 2 was shown in the 26‐week HELP study (NCT02586805). Eligible patients were aged ≥12 years old and had ≥1 investigator‐confirmed HAE attacks per month; patients randomized to lanadelumab 300 mg SC every 2 weeks had an 87% reduction in monthly HAE attack rate compared with those randomized to placebo (mean rate ratio: 0.13 [95% CI, 0.07–0.24]; *p* < 0.001).^37^ Exploratory findings from ad hoc analyses from the HELP study found onset of attack prevention within 2 weeks of lanadelumab initiation and sustained attack prevention throughout the 26‐week study.^38^ The long‐term open‐label extension of the HELP study (median duration 33 months) reported sustained efficacy with no new safety concerns.^39^, ^40^

Another plasma kallikrein inhibitor, berotralstat (ORLADEYO™; BioCryst Pharmaceuticals, Inc.) was approved as prophylaxis to prevent attacks of HAE in adults and pediatric patients aged ≥12 years.^41^, ^42^ The efficacy of berotralstat was demonstrated in the 24‐week randomized phase 3 APeX‐2 study (NCT03485911) of patients with type 1 or 2 HAE who experienced ≥2 investigator‐confirmed HAE attacks requiring treatment or causing functional impairment in the first 56 days of the prospective run‐in period. Berotralstat 150 mg once daily significantly reduced attacks relative to placebo: 1.31 attacks per month versus 2.35 attacks per month (*p* < 0.001).^43^ APeX‐2 was designed as a 3 part study, and the results of Part 2, which evaluated an additional 24 weeks of blinded berotralstat treatment without a control group, were recently reported.^44^ Mean attack rates among patients receiving berotralstat 150 mg/day declined by 67% from baseline to week 48; reductions in attack rates either continued or declined further from Part 1 to Part 2 of the study, with no new safety signals. Part 3 of Apex‐2, a long‐term open‐label extension phase, is ongoing.

## 3 GUIDELINES REGARDING LTP OF HAE

A comprehensive and effective HAE management plan is intended to help “normalize” a patient's life as much as possible, so that they can fully engage in work, school, family, and leisure activities.^13^ Although on‐demand treatment is effective, for many patients it may be inadequate. Novel therapies for LTP are now available (Table 1) and are anticipated to shift the management paradigm to increased adoption of LTP, which offers the benefit of reducing the number and intensity of attacks, and thereby can improve quality of life.

**TABLE 1:** HAE pathway‐specific options approved for LTP

	Route of administration	FDA approved indication for LTP
C1INH replacement therapies		
C1INH [human] (CINRYZE^®^; Shire/ViroPharma Inc.; Lexington, MA)^17^, ^18^	IV	Routine prophylaxis against angioedema attacks in adults, adolescents, and pediatric patients (6 years of age and older) with HAE
C1INH [human] (HAEGARDA^®^ [US]; Berinert 3000 [EU])^®^; CSL Behring GmBH; Marburg, Germany)^23^, ^24^	SC	Routine prophylaxis to prevent HAE attacks in patients 6 years of age and older (United States) or “adolescent” and older (EU)
Plasma kallikrein inhibitors		
Lanadelumab‐flyo (TAKHZYRO™; Dyax; Lexington, MA)^35^, ^36^	SC	Prophylaxis to prevent attacks of HAE in patients 12 years and older
Berotralstat (ORLADEYO™; BioCryst Pharmaceuticals, Inc., Durham, NC)^41^, ^42^	Oral	Prophylaxis to prevent attacks of HAE in adults and pediatric patients 12 years and older

Abbreviations: C1INH, C1 inhibitor; HAE, hereditary angioedema; IV, intravenous; LTP, long‐term prophylaxis; SC, subcutaneous.

The World Allergy Organization (WAO), in collaboration with the European Academy of Allergy and Clinical Immunology (EAACI), recommends that all patients with HAE be evaluated for LTP routinely.^11^ The recently released HAE Association (HAEA) guidelines note that the decision to use LTP should not be based on rigid criteria, but rather should be based on an individual patient's needs.^13^ Factors related to disease burden as well as the patient's quality of life and treatment preference should be evaluated.^13^, ^45^ Key decision factors when considering LTP are noted in Table 2 **.**

**TABLE 2:** Key decision factors in considering and individualizing LTP^12^, ^13^

Clinical aspects of disease	Quality of life impact	Health‐system and treatment‐related factors
Overall disease burden/comorbidities Angioedema attack frequency Prior severe, debilitating, or life‐threatening attacks	Missed work or school Interference with event planning (e.g., vacations, family occasions). Inability to conduct ADL Fear and anxiety about future attacks	Access to urgent care Benefit‐risk and/or treatment burden of available HAE management options

Abbreviations: ADL, activities of daily living (e.g., holding utensils and implements, walking, driving, exercising, childcare, and/or eldercare); HAE, hereditary angioedema; LTP, long‐term prophylaxis.

Both the WAO/EAACI and HAEA guidelines recommend C1INH as first‐line therapy for LTP. Dosage and/or treatment interval for LTP should be adapted, as needed, to minimize disease burden.^13^, ^45^ The HAEA also recommends the kallikrein inhibitor, lanadelumab, as a first‐line option for LTP.^13^ HAE pathway‐specific treatments for LTP are discussed in detail above.

Androgens are a second‐line option for LTP that may be used in special circumstances (e.g., if the recommended first‐line pathway‐based options are unavailable, or if a patient not willing/able to use recommended injectable treatment).^13^ As noted previously, with the increased use of pathway‐specific treatments, particularly C1INH, use of androgens for HAE has declined substantially in the past decade.^8^ Use of androgens for LTP is limited by poor tolerability; moreover, these agents are not recommended in children and are contraindicated during pregnancy.^45^, ^46^, ^47^

In contrast to older agents for LTP, such as androgens that are limited by dose‐related side effects, newer agents are not associated with notable safety concerns, with the most common adverse events being transient local site reactions with C1INH(SC) and site reactions or dizziness with lanadelumab.^13^

A summary of the updated WAO/EAACI and HAEA guidelines regarding LTP in adults, children/adolescents, as well as in pregnant/lactating patients is provided in Table 3.^13^, ^45^ Notably, C1INH is recommended as first‐line therapy for LTP for each of these patient populations. The WAO/EAACI and HAEA recommendations are consistent with pediatric‐specific guidelines, which consider C1INH to be a preferable and safe option for LTP in children.^6^, ^48^

**TABLE 3:** HAE guideline recommendations regarding LTP (A: WAO/EAACI guidelines^45^); (B: HAEA guidelines^13^)

A.	
WAO/EAACI recommendations regarding LTP	
Consideration of LTP	
	Recommend that all patients (adult and pediatric) be evaluated for LTP at every visit; disease burden and patient preference should be taken into consideration (grade of evidence: D; strength of recommendation: Strong, 100% agreement).
	LTP may be indicated during pregnancy, especially in patients who experience an increase in the frequency of HAE attacks.
Pharmacological management of LTP	
Adults	Recommend use of C1INH for first‐line LTP (grade of evidence: A; strength of recommendation: Strong, 50%–75% agreement [majority vote]).
	Suggest use of androgens as second‐line LTP (grade of evidence: C; strength of recommendation: Weak, 50%–75% agreement [majority vote]).
	Suggest adaptation of LTP (e.g., dosage and/or treatment interval) as needed to minimize disease burden (grade of evidence: D; strength of recommendation: Weak, 100% agreement).
Children/adolescents	Plasma‐derived C1INH is the preferred therapy for LTP; dosing interval and dose of C1INH may need to be adjusted according to the individual response.
	When C1INH concentrate is not available for LTP, antifibrinolytics are preferred over androgens because of their better safety profile; however, efficacy is questionable and data supporting use are not available.
	Androgens are not recommended in children and adolescents prior to Tanner Stage V; however, long‐term use has been reported, and in some cases the benefits may outweigh the risks; administration of androgens requires careful safety monitoring.
Pregnant/lactating patients	C1INH concentrate is considered a safe and effective treatment option.
	Antifibrinolytics may be considered if C1INH concentrate is unavailable, but efficacy is not proven.
	Androgens are contraindicated, as these drugs cross the placenta (adverse effects include masculinization of the female fetus, placental insufficiency, and fetal growth retardation).
	Breastfeeding should be discontinued before androgens are introduced; terminating lactation itself may reduce attack frequency.

B.	
HAEA recommendations regarding LTP	
Consideration of LTP	
	The decision on when to use LTP cannot be made on rigid criteria but should reflect the needs of the individual patient (level of evidence: High for HAE C1INH; low for HAE‐nl‐C1INH; strength of recommendation: Strong).
Pharmacological management of LTP	
Adults	LTP treatment of HAE‐C1INH should include first‐line medications (C1INH [IV], C1INH [SC], or lanadelumab; level of evidence: High; strength of recommendation: Strong).
Children/adolescents	Indications for the use of first‐line HAE medications are the same in children as in adults, although regulatory differences affect the use of some medications depending on the child's age (level of evidence: Moderate; strength of recommendation: Strong).
Pregnant/lactating patients	C1INH is the recommended HAE medication for use as either on‐demand or prophylactic therapy (level of evidence: Moderate; strength of recommendation: Strong).

Abbreviations: C1INH, C1 inhibitor; EAACI, European Academy of Allergy and Clinical Immunology; HAE, hereditary angioedema; HAEA, Hereditary Angioedema Association; IV, intravenous; LTP, long‐term prophylaxis; SC, subcutaneous; WAO, World Allergy Organization.

## 4 CONSIDERATIONS FOR CLINICAL PRACTICE

Allergists and other healthcare providers have an ongoing commitment to discuss management options with their HAE patients, reassess regularly, and make adjustments.^49^ Patient preference is a central consideration, so while healthcare providers should not necessarily push for LTP, it is important to inform patients that there are effective new drugs available, especially for patients who express concerns about “lack of control” of their disease. For example, LTP should be considered for patients who are missing school/work and/or are limiting recreational activities/travel–essentially contracting their lifestyle in order to reduce triggers and fit within the domain of their disease. In contrast, LTP would not be an ideal fit if a patient considers the associated treatment burden to be greater than their disease burden.

A periodic review of the nature and frequency of attacks, triggers, and treatments provides the opportunity to identify and address any needed changes to the management plan for HAE.^49^ The updated HAE guidelines emphasize the importance of an ongoing dialogue regarding LTP. Routinely asking patients about LTP, rather than assuming that an existing HAE management plan remains adequate, enables healthcare providers to stay current with their patient's potentially changing needs.

Likewise, this type of proactive engagement encourages the patient to reevaluate their own lifestyle and goals in relation to their disease and ask questions about new therapies.^50^, ^51^, ^52^ Due to both the variability in the disease and changes in the patient's circumstances and/or preferences, the threshold for/barriers to LTP may change over time. An ongoing and iterative shared “3D” decision‐making model for provider‐patient collaboration in HAE has been proposed, with the 3D's being “discover“ (i.e., explore patient's needs/preferences, establish goals, acknowledge available options), “discuss” (i.e., discuss reasonable alternatives aligned to the patient's needs/preferences), and “decide” (i.e., make shared decisions based on mutual understanding and accurate information).^50^ Shared decision‐making, along with routine monitoring, allows for informed and timely adjustments of the management plan, if needed.^51^ Beyond choice of on‐demand treatment and LTP, shared decision‐making is also useful in other aspects of HAE management, such as genetic testing and screening of family members.^52^

The choice of LTP should be individualized.^53^ C1INH and lanadelumab are generally the preferred options for LTP. Treatment‐related burdens and barriers should be considered and addressed when adapting management to patient needs. For example, many patients using C1INH(IV) for LTP experience challenges related to IV access.^54^ While indwelling ports may address the issue of IV access, associated risks include blockage, thrombosis, and infection.^54^, ^55^, ^56^ Alternate routes of administration, including SC and oral options for LTP, may offer convenience and can facilitate self‐administration, and thus may improve adherence.

C1INH products have been used for over 40 years globally and are the standard of care for LTP of HAE. C1INH can be used across a range of patient groups, including children as young as 6 years old (United States) or “adolescents” (EU), and in women who are of childbearing age, pregnant, or lactating.^23^, ^24^, ^57^, ^58^, ^59^ Regarding use in the elderly, the US prescribing information notes that “clinical experience has not identified differences in responses between the elderly and younger patients,” but recommends that dosing be initiated at the low end of the dosing range, since this population has the potential for decreased hepatic, renal, or cardiac function as well as potential for more concomitant disease and/or other drug therapy.^24^ Finally, patients with HAE should be made aware of patient advocacy organizations, such as the HAE international (HAEi) and US HAEA, as these provide important sources of patient support and education about their disease and management options, including LTP.^60^, ^61^ For example, HAEi offers an electronic diary app (HAE TrackR) for tracking attacks, treatments, and life impact—a report can be generated from these data and serve as an HAE management tool for patients/caregivers and physicians.^62^

## 5 CONCLUSIONS

Assessment of a patient's need for LTP is an important part of the ongoing dialogue between providers and patients living with HAE, as both disease‐related factors and patient preferences may change over time. LTP can be an instrumental part of an HAE management plan that enables patients to feel “normalized,” and therefore be fully engaged in their work/school, family, and leisure activities, rather than limited by the constraints of uncontrolled disease. Plasma‐derived C1INH is the broadly recommended first‐line option for LTP in patients with HAE, including pregnant and/or lactating women, and pediatric patients aged as young as 6 years.

## CONFLICT OF INTEREST

John Anderson has served as a speaker for, has been on advisory boards for, and has worked on clinical research with Pharming, CSL Behring, Shire/Takeda, and BioCryst; he has been on an advisory board for BioMarin. Njeri Maina reports no conflicts of interest.
